# Dietary Carotenoid Intakes and Prostate Cancer Risk: A Case-Control Study from Vietnam

**DOI:** 10.3390/nu10010070

**Published:** 2018-01-11

**Authors:** Dong Van Hoang, Ngoc Minh Pham, Andy H. Lee, Duong Nhu Tran, Colin W. Binns

**Affiliations:** 1 National Institute of Hygiene and Epidemiology, Hanoi 100000, Vietnam; trannhuduong@gmail.com; 2 School of Public Health, Curtin University, Perth, WA 6102, Australia; minh.pn@tnu.edu.vn (N.M.P.); andy.lee@curtin.edu.au (A.H.L.); c.binns@curtin.edu.au (C.W.B.); 3 Thai Nguyen University of Medicine and Pharmacy, Thai Nguyen 250000, Vietnam

**Keywords:** case-control study, carotenoids, epidemiology, prostate cancer, Vietnam

## Abstract

The incidence of prostate cancer has increased in Vietnam, but there have been few studies of the risk factors associated with this change. This retrospective case-control study investigated the relation of the intake of carotenoids and their food sources to prostate cancer risk. A sample of 652 participants (244 incident prostate cancer patients, aged 64–75 years, and 408 age frequency-matched controls) were recruited in Ho Chi Minh City during 2013–2015. The habitual diet was ascertained with a validated food-frequency questionnaire, and other factors including demographic and lifestyle characteristics were assessed via face-to-face interviews by trained nurses. Multivariate-adjusted odds ratios (OR) and 95% confidence intervals (CI) were estimated using unconditional logistic regression models. The risk of prostate cancer decreased with increasing intakes of lycopene, tomatoes, and carrots; the respective ORs (95% CIs) were 0.46 (0.27, 0.77), 0.39 (0.23, 0.66), and 0.35 (0.21, 0.58), when comparing the highest with the lowest tertile of intake (*p* for trend < 0.01). No statistically significant associations were found for the intake of α-carotene, β-carotene, β-cryptoxanthin, lutein, zeaxanthin, and major food sources of carotenoids. In conclusion, Vietnamese men with a higher intake of lycopene, tomatoes, and carrots may have a lower risk of prostate cancer. However, large prospective studies are needed in this population to confirm this finding.

## 1. Introduction

Prostate cancer is globally a common malignancy for males [1]. In developing countries, despite its relatively low prevalence (<20 per 100,000), prostate cancer has shown an increasing trend in recent decades [2]. A healthy diet may play a role in preventing the development of prostate cancer [3]. Accumulated data from epidemiological studies suggested that eating more fruits and vegetables may reduce the risk of prostate cancer [4]. Plant carotenoids, especially carotene and lycopene, are phytochemicals which contribute to that inverse association [5,6,7]. There are multiple mechanisms which may be involved in the bioactivity of these compounds in relation to prostate cancer development, including protecting the DNA from free radicals [8] and modulating gene expression [9].

However, the epidemiological evidence for the association between the intake of specific carotenoids and the risk of prostate cancer remains inconsistent [10,11,12]. The most commonly studied carotenoids for a potential protective benefit against prostate cancer are lycopene, α-carotene, β-carotene, β-cryptoxanthin, lutein, and zeaxanthin [13]. Among them, lycopene has received the most attention and supportive evidence, followed by β-carotene, while the evidence for other carotenoids is still inconclusive [14,15]. Studies on the relationship between carotenoid intake and prostate cancer risk have largely been conducted in North American and European countries, and corresponding data is scarce in Asian nations [12,15]. It is important to discern whether dietary intakes of carotenoid and their major sources are associated with the risk of prostate cancer among populations with low or middle income, including Vietnamese men who experienced an upward trend in prostate cancer incidence over the past decade [16]. The objective of this paper is to report findings of a case-control study that investigated the association of dietary intakes of major carotenoids and their food sources with the risk of prostate cancer in Vietnam.

## 2. Materials and Methods

A case-control study of 652 participants was conducted in Ho Chi Minh City, Vietnam, during 2013 and 2015. The cases were histologically confirmed prostate cancer patients, aged 64–75 years, with the diagnosis made within one week before their interview. Of the 272 eligible incident patients, 253 agreed to participate and were consecutively recruited and interviewed. Subsequently, nine patients were excluded because of either missing or implausible information, leaving 244 prostate cancer patients for subsequent analysis.

Eligible controls were men who were residing in the same catchment area as the cases and did not report having any severe or malignant condition, or attended the same hospitals for minor health problems (e.g., oral health conditions and minor injuries). They were frequency-matched to the cases by 5 years age groups. Of a total of 700 male residents who were contacted, 429 men consented to participate in the study. However, 62 participants were excluded because of: (1) refusal to provide a blood sample for the prostate specific antigen (PSA) test, or having over 4 ng/mL serum PSA; (2) having malignant or severe chronic diseases. As a result, 367 eligible men were recruited and interviewed. In addition, 120 eligible patients who attended the same hospitals (as the cases) for a health check or for treatment of minor health issues were also approached. Eighty-three of these men gave their consent for the interview. Due to implausible or missing information, a further 28 community-based controls and 14 hospital-based controls were excluded. Finally, 244 cases and 408 controls (339 community-based and 69 hospital-based) were included in the statistical analysis.

The collected data consisted of information about dietary intakes, demographic, and lifestyle characteristics, and were obtained through face-to-face interviews using a structured questionnaire. Each interview took about 40 min to complete. In order to maximize the accuracy of the information provided, the participant’s next-of-kin were encouraged to participate in the interview.

An information sheet was provided to all participants, and written consent was obtained for every interview. Both interviewers and participants were blinded to the study hypothesis. This study was approved by the Human Research Ethics Committee of Curtin University (approval number: HR 109/2012).

The dietary intake was assessed using an 89 item semi-quantitative food frequency questionnaire validated in Vietnamese adults [17], with only minor textual modifications. The recall period for dietary habits was set to three years before the interview. A picture booklet was used to assist participants in estimating the intake amount and portion size of certain food items. Tomato sauce was excluded because it was not commonly used in Vietnam. We estimated intakes (μg/day) of lycopene, α-carotene, β-carotene, β-cryptoxanthin, lutein, and zeaxanthin using the US Department of Agriculture nutrient database [18] because of insufficient data provided by the current Vietnamese Food Composition Tables, except for energy intake (kcal/day). We applied the residual method to adjust for variations in energy intake from carotenoid nutrients [19].

Other exposure measurements included demographic and lifestyle characteristics (e.g., age, marital status, education level, and smoking), medical history (including histological examination of the prostate gland and PSA levels), height, and weight. The questions on life-long physical activity exposure was taken from the study “Life-long physical activity involvement and the risk of ischemic stroke in southern China” [20]. The questions on smoking and alcohol drinking were based on the WHO STEPwise approach to noncommunicable disease risk factor surveillance [21].

The distributions of the study variables were examined by case-control status. Demographic and lifestyle characteristics of the participants, as well as carotenoid intake variables, were compared between case and control groups using a two-sample *t*-test for continuous variables, and a chi-square test for categorical variables. Because the cases and controls were not individually matched, unconditional logistic regression analyses were performed to ascertain the strength of the association between intakes of carotenoids and major food sources and prostate cancer risk.

Independent variables (i.e., carotenoids) were transformed into categorical variables based on tertile distribution of controls [22]. The lowest level of each food and nutrient intake was treated as the reference category. Crude and adjusted odds ratios (OR) and associated 95% confidence intervals (CI) were reported. Tests for linear trend were performed with logistic regression models by assigning an ordinal value (i.e., 1–3) to each tertile of the intake of carotenoids and their food sources in relation to the risk of prostate cancer.

Confounding variables included in the logistic regression models were education level (primary, high, and tertiary), marital status (married, never married, or separated), smoking habit (never, former, current), presence of prostate cancer in the first-degree relatives (yes, no), life-long physical activity (never, past active, regular), age (year, continuous), body mass index (kg/m^2^, continuous), ethanol consumption (g/day, continuous), and total energy intake (kcal/day, continuous). These variables were either established or plausible risk factors according to the literature. Additionally, the cases were categorized into low-medium- (Gleason score ≤ 7) and high-grade (Gleason score 8–10) prostate cancer [23] for subgroup analyses. Tests for heterogeneity were performed to evaluate whether the associations between the intake of carotenoids and their food sources and prostate cancer differed by the grade of the tumors. For these analyses, we separately fitted two multivariate logistic regression models for low-medium-grade and high-grade tumors, and compared the risk coefficients and standard errors in each subgroup [24]. The *p*-values were obtained from Cochran’s Q statistic with a χ^2^ distribution and one degree of freedom [25].

All statistical analyses were performed using the *R* Statistics software version 3.3.3 [26]. A *p*-value < 0.05 was considered statistically significant.

## 3. Results

The sample consisted of 408 controls and 244 cases, and the average age of the participants was 68 years. Compared to the controls, the prostate cancer patients were married at a younger age, had fewer children, smoked more tobacco cigarettes, and drank more alcohol, and they had a significantly lower energy intake before the diagnosis. The two groups also differed in terms of educational level and lifetime physical activity, with the cases being less educated and less active than their control counterparts. A first-degree family history of prostate cancer was reported by only seven cases (Table 1).

Table 2 shows that prostate cancer patients reported significantly lower consumption levels of carotenoids than controls (*p* < 0.05). Similarly, the intake levels of the major sources of carotenoids (e.g., tomato and carrot) were also significantly lower among cases when compared to the controls (*p* < 0.001).

The results of the logistic regression analyses showed that increasing intakes of lycopene, tomato, and carrot were significantly associated with a lower risk of prostate cancer (Table 3). Specifically, the adjusted OR for prostate cancer was 0.46 (95% CI: 0.27, 0.77) when comparing the highest versus the lowest tertile of lycopene intake. Similarly, for the intake of tomato and carrot, the adjusted ORs for prostate cancer were 0.39 (95% CI: 0.23, 0.66) and 0.35 (95% CI: 0.21, 0.58), respectively. These inverse associations were fully accounted for potential covariates, namely education level, marital status, smoking habit, family history of prostate cancer, life-long physical activity, age, body mass index, ethanol consumption, and total energy intake. For other exposures with seemingly increased risk in the second tertile of intake, their *p*-values when tested for linear trend were not significant, so no conclusion can be drawn. Results remained unchanged in either low-medium- or high-grade prostate cancers (See Appendix A).

## 4. Discussion

To the best of our knowledge, this is the first epidemiological study to examine the association between the dietary intake of carotenoids and their major food sources and prostate cancer in the Vietnamese population. In this case-control study, we found that higher intakes of lycopene, tomato, and carrot were significantly associated with a reduced risk of prostate cancer. The inverse associations we observed were dose-responsive and independent of factors commonly associated with prostate cancer, including age, family history of prostate cancer, and body mass index. However, there was a lack of significant association between other dietary carotenoids and foods rich in these phytochemicals and prostate cancer risk.

The present data regarding the intake of lycopene is similar to the results of previous studies. A recent meta-analysis of 42 studies showed that participants with the highest level of lycopene intake or blood lycopene had a 12% significantly lower risk of prostate cancer [12] as compared to individuals with the lowest level. It should be noted that the inverse association between lycopene and prostate cancer risk observed in that meta-analysis was restricted to case-control studies and was not observed in prospective studies, consistent with the study by Key et al. [5]. Similarly, another meta-analysis including 34 studies reported an inverse association between lycopene intake or blood levels and the risk of prostate cancer, with a stronger association observed for the circulating lycopene (OR: 0.81; 95% CI: 0.69–0.96) when comparing the highest to the lowest level [15]. Although this study indicated an inverse association between lycopene and prostate cancer risk, large prospective cohort studies with biological samples are needed to confirm our findings because of the lack of conclusive evidence for the lycopene–prostate cancer relationship in the literature.

There are some possible pathways through which lycopene may protect against prostate cancer. The antioxidant properties of lycopene can prevent DNA damage by scavenging free radicals [8]. Lycopene can also modulate gene expression related to prostate cancer growth [9,27] and slow cancer cell growth [28]. It has been demonstrated that lycopene inhibits prostate cancer cell proliferation via the PPARγ-LXRα-ABCA1 pathway [29]. Additionally, this non-provitamin A carotenoid was shown to hamper the progression of prostate cancer via apoptosis induction and angiogenesis suppression [30,31].

Tomato is one of the major sources of lycopene [32,33], and the lower risk of prostate cancer observed among men with higher intake of tomato reported here is consistent with previous studies conducted among Western and Asian populations. Pooled data from seven reports in Asia, namely in China, Japan, Malaysia, and Iran, found a strong, inverse association between tomato intake and the risk of prostate cancer (the summary relative risk: 0.43; 95% CI: 0.22–0.85) [34]. Likewise, cooked tomato intake has been shown to decrease prostate cancer risk in a meta-analysis of 21 studies (pooled RR: 0.81; 95% CI: 0.71–0.92) [35]. The mechanism underlying the beneficial effect of dietary tomato on prostate cancer risk can be explained by the high biological activity of lycopene found in tomato (see discussion above). Another possibility is the anticancer effect of phenolic compounds [36] that accounts for the considerable antioxidant properties of tomato [37,38,39,40,41]. Given the popularity of tomatoes in Asia, further evidence from longitudinal studies is required to help an individual make an informed choice perform an informed choice regarding the consumption of tomato for prostate cancer prevention.

Carrots (dacus carrota) are commonly consumed worldwide, particularly in Western countries, and are a rich source of α-carotene and β-carotene [36]. Although the consumption of carrots in our study was low compared with others [42], we found a strong dose-responsive inverse association with prostate cancer. Consistent with this finding, a recent meta-analysis including 10 studies (eight case-control and two cohort) showed around 20% significantly lower risk of prostate cancer in the category with the highest level of carrot consumption relative to the one with the lowest level [43]. Because of the scarcity of studies on dietary carrots and prostate cancer risk in Asia [43], our study adds to the evidence for a potentially beneficial role of carrot in prostate cancer prevention among populations, even with a relatively low consumption.

The observed inverse association between carrot intake and prostate cancer may be attributable to the bioavailability of carotene abundantly present in carrot [44]. However, neither α-carotene nor β-carotene was related to prostate cancer risk in the present study. The lack of the association between β-carotene and prostate cancer risk is consistent with a recent meta-analysis of 34 studies that found null findings for dietary β-carotene (including 19 studies: the pooled RR: 0.90; 95% CI: 0.81–1.01) and circulating β-carotene (comprising 13 studies: the pooled RR: 0.96; 95% CI: 0.81–1.14) when comparing the highest versus the lowest level of β-carotene [15]. Another individual participant data meta-analysis of 15 studies also demonstrated no statistically significant associations between blood carotene levels and overall prostate cancer risk [5]. Regarding α-carotene, there are inconsistent findings in the available literature on its association with prostate cancer risk. Although the pooled result of 12 studies showed an inverse association between dietary α-carotene intake and overall risk of prostate cancer [15], only two studies [45,46] included in that meta-analysis found a significantly lower risk of prostate cancer in individuals with higher α-carotene intake. Moreover, accumulating data have suggested no evidence for the association between circulating α-carotene and prostate cancer risk [5,15]. Given this, there is a need for conducting long-term clinical trials to elucidate the role of carotene in the development and progression of prostate cancer.

β-cryptoxanthin is the major carotenoid in several tropical orange-fleshed fruits and is abundantly found in oranges, tangerines, red pepper, papaya [18]. The predominant sources of lutein and zeaxanthin are spinach, broccoli, brussels sprout, squash, green beans, sweet corn, kale, and lettuce [18]. It has been suggested that these carotenoids have anticarcinogenic activity [47,48]. Nonetheless, similar to dietary carotene intake, we observed no relation between the intake of β-cryptoxanthin as well as lutein and zeaxanthin and prostate cancer risk. Studies reported in the literature are inconclusive with respect to the association between dietary intakes of β-cryptoxanthin, together with lutein and zeaxanthin, and prostate cancer risk. Some studies reported an inverse association for β-cryptoxanthin [45,49,50] or lutein and zeaxanthin [45,50,51], whereas others found null results [51,52,53,54,55]. Moreover, recent pooled findings of 15 individual studies showed that circulating β-cryptoxanthin, lutein, and zeaxanthin were not associated with the risk of prostate cancer (respective summary OR and 95% CI: 0.92 [0.82–1.03], 0.98 [0.86–1.12] and 1.08 [0.92–1.26], comparing the highest versus the lowest levels) [5]. The absence of the association of dietary intakes of beta-cryptoxanthin, lutein, and zeaxanthin, and other food sources (i.e., pumpkin, sweet potato, water melon, and citrus fruits) with the risk of prostate cancer requires confirmation in future studies.

There are several limitations to be considered when interpreting the results of the present study. First, a cause–effect relationship between dietary lycopene and its major sources and the risk of prostate cancer cannot be established because of the retrospective cross-sectional design. Second, there are inherent biases for this observational study. A selection bias may have been present as participants were voluntary and not randomly selected from the population. Additionally, controls included some participants who had dental issues, which may have affected food consumption. An information bias, however, was unlikely because all participants were unaware of the study hypothesis, while the role of dietary carotenoids in the development of prostate cancer has not been confirmed, particularly in Vietnam. A recall bias may occur if the cases recalled their history of dietary habits differently from the controls. To minimize the bias and to improve the accuracy of the information obtained, we employed the same well-trained interviewers to conduct direct interviews of both case and control groups using an identical protocol under similar conditions. Information about dietary habit of the study subjects was also sought from the participant’s next-of-kin. Third, we did not measure carotenoids in blood to consolidate the results from the dietary intake; however, the reported correlations between dietary and plasma carotenoids from a recent meta-analysis of 142 studies [56] are weak to moderate (0.26–0.47). Although all selected controls had a PSA level ≤4 ng/mL, a misclassification of their case-control status would still be possible. Indeed, the resulting association should have been weakened, given the low incidence of prostate cancer in Vietnam [57]. Another limitation is that we had only data on the grade of the cancer but not on its stage, and the sample size was not large, which may affect subgroup analyses. Finally, all participants were recruited from the same catchment area within Ho Chi Minh City, and our findings may not be generalizable to the entire Vietnamese population.

## 5. Conclusions

This case-control study showed an inverse, dose-response association of dietary lycopene, tomato, and carrot with prostate cancer risk among Vietnamese men, despite the lack of association for other dietary carotenoids (i.e., α-carotene, β-carotene, β-cryptoxanthin, as well as lutein and zeaxanthin) and their food sources. Our results add to the evidence for the potentially beneficial role of eating foods rich in lycopene as a possible protection against prostate cancer in Asia. However, the replication of the present study in other locations and a large prospective study in this population would assist in confirming the findings.

## Figures and Tables

**Table 1:** Sample characteristics.

Demographic Variable	Case (*n* = 244)	Control (*n* = 408)	*p*-Value ^†^
Age (year), mean (SD)	68.7 (7.3)	68.0 (5.8)	0.155
Age at marriage (year), mean (SD)	25.0 (4.9)	27.3 (4.9)	<0.001
Body mass index (kg/m^2^), mean (SD)	22.0 (3.0)	21.9 (3.3)	0.913
Waist to hip ratio, mean (SD)	0.9 (0.1)	0.9 (0.1)	0.128
Smoking habit (pack, years), mean (SD)	16.2 (20.2)	12.8 (14.7)	0.025
Ethanol (g/day), mean (SD)	22.6 (47.5)	14.9 (28.4)	0.021
Total energy (kcal/day), mean (SD)	1712.0 (642.0)	2101.0 (832.0)	<0.001
Number of children, *n* (%)			
≤3	103 (42.2)	223 (54.7)	
4–6	92 (37.7)	148 (36.3)	<0.001
≥7	49 (20.1)	37 (9.0)	
Education level, *n* (%)			
Primary school	65 (26.6)	72 (17.6)	0.019
High school	134 (54.9)	261 (64.0)	
Tertiary education	45 (18.4)	75 (18.4)	
Marital status, *n* (%)			
Married	233 (95.5)	375 (91.9)	0.109
Never married or separated	11 (4.5)	33 (8.1)	
Smoking history, *n* (%)			
Never	58 (23.8)	111 (27.2)	0.182
Former	121 (49.6)	172 (42.2)	
Current	65 (26.6)	125 (30.6)	
Prostate cancer in the first-degree relatives (yes), *n* (%)	7 (2.9)	0 (0.0)	0.002
Life-long physical activity, *n* (%)			
Never	200 (82.0)	192 (47.1)	<0.001
Past active	25 (10.2)	102 (25.0)	
Regular	19 (7.8)	114 (27.9)	

^†^ *p*-value from *t*-Test or chi-square test. SD: standard deviation.

**Table 2:** Comparison of the intakes of carotenoids and their major sources between cases and controls.

Nutrient or Food	Case (*n* = 244)	Control (*n* = 408)	*p*-Value ^†^
Lycopene (μg/day), mean (SD)	839.6 (1087.2)	1356.2 (1527.9)	<0.001
α-carotene (μg/day), mean (SD)	756.5 (294.7)	919.8 (411.3)	<0.001
β-carotene (μg/day), mean (SD)	4473.4 (2555.0)	5491.9 (3472.0)	<0.001
β-cryptoxanthin (μg/day), mean (SD)	749.2 (420.9)	839.6 (557.8)	0.019
Lutein and zeaxanthin (μg/day), mean (SD)	2147.7 (1325.7)	2531.9 (1625.0)	0.001
Tomato (g/day), mean (SD)	10.9 (21.2)	19.6 (28.5)	<0.001
Carrot (g/day), mean (SD)	1.6 (2.7)	3.6 (6.5)	<0.001
Pumpkin (g/day), mean (SD)	15.1 (16.5)	16.9 (18.4)	0.193
Sweet potato (g/day), mean (SD)	8.0 (17.1)	11.5 (27.7)	0.042
Watermelon (g/day), mean (SD)	14.5 (19.5)	19.5 (37.9)	0.028
Citrus fruits (g/day), mean (SD)	6.1 (17.2)	9.2 (22.2)	0.048

^†^ *p*-value from *t*-Test. SD: standard deviation.

**Table 3:** Crude and adjusted odds ratios and associated 95% confidence intervals of prostate cancer risk for the intakes of carotenoid and their sources.

Nutrient or Food	Mean Intake (SD)	Case, *n* (%)	Control, *n* (%)	OR (95% CI)	Adjusted OR ^†^ (95% CI)	*p* for Linear Trend ^‡^
Lycopene (μg/day)						
<648	388.9 (109.2)	77 (31.6)	136 (33.3)	1.00 reference	1.00 reference	
648–1200	809.6 (184.1)	124 (50.8)	137 (33.6)	1.60 (1.11, 2.32)	0.83 (0.52, 1.34)	0.003
>1200	2810.5 (1918.6)	43 (17.6)	135 (33.1)	0.56 (0.36, 0.87)	0.46 (0.27, 0.77)	
α-carotene (μg/day)						
<743	547.7 (135.1)	89 (36.5)	136 (33.3)	1.00 reference	1.00 reference	
743–976	861.7 (82.7)	105 (43.0)	136 (33.3)	1.18 (0.82, 1.71)	1.19 (0.78, 1.83)	0.307
>976	1336.9 (367.7)	50 (20.5)	136 (33.3)	0.56 (0.37, 0.85)	0.77 (0.47, 1.26)	
β-carotene (μg/day)						
<3920	2841.3 (601.1)	74 (30.3)	137 (33.6)	1.00 reference	1.00 reference	
3920–5780	4672.6 (625.9)	120 (49.2)	135 (33.1)	1.65 (1.13, 2.40)	1.41 (0.91, 2.19)	0.248
>5780	8904.4 (3828.9)	50 (20.5)	136 (33.3)	0.68 (0.44, 1.04)	0.73 (0.44, 1.22)	
β-Cryptoxanthin (μg/day)						
<539	404.8 (102.9)	62 (25.4)	137 (33.6)	1.00 reference	1.00 reference	
539–867	705.1 (93)	100 (41.0)	135 (33.1)	1.64 (1.10, 2.44)	1.63 (1.03, 2.60)	0.303
>867	1388.5 (550.8)	82 (33.6)	136 (33.3)	1.33 (0.89, 2.00)	1.29 (0.79, 2.09)	
Lutein and zeaxanthin (μg/day)						
<1670	1234.6 (293.3)	69 (28.3)	137 (33.6)	1.00 reference	1.00 reference	
1670–2580	2081.7 (272.9)	122 (50.0)	135 (33.1)	1.79 (1.23, 2.63)	1.50 (0.97, 2.34)	0.223
>2580	4269.3 (1708.0)	53 (21.7)	136 (33.3)	0.77 (0.50, 1.19)	0.73 (0.44, 1.20)	
Tomato (g/day)						
<7.1	3.1 (2.1)	87 (35.7)	136 (33.3)	1.00 reference	1.00 reference	
7.1–16.5	10.6 (3.4)	114 (46.7)	136 (33.3)	1.31 (0.91, 1.89)	0.68 (0.43, 1.06)	<0.001
>16.5	47.9 (39.6)	43 (17.6)	136 (33.3)	0.49 (0.32, 0.76)	0.39 (0.23, 0.66)	
Carrot (g/day)						
<1	0.4 (0.3)	111 (45.5)	139 (34.1)	1.00 reference	1.00 reference	
1–3.2	2 (0.7)	94 (38.5)	132 (32.4)	0.89 (0.62, 1.28)	0.61 (0.39, 0.94)	<0.001
>3.2	7.8 (8.7)	39 (16.0)	137 (33.6)	0.36 (0.23, 0.55)	0.35 (0.21, 0.58)	
Pumpkin (g/day)						
<6.4	3.3 (2.1)	69 (28.3)	138 (33.8)	1.00 reference	1.00 reference	
6.4–20.5	12 (3.6)	101 (41.4)	135 (33.1)	1.50 (1.02, 2.21)	1.01 (0.64, 1.59)	0.423
>20.5	34.5 (19.3)	74 (30.3)	135 (33.1)	1.10 (0.73, 1.64)	0.82 (0.51, 1.32)	
Sweet potato (g/day)						
<2.4	0.2 (0.3)	51 (20.9)	135 (33.1)	1.00 reference	1.00 reference	
2.4–7.7	2.3 (0.9)	76 (31.1)	138 (33.8)	1.46 (0.95, 2.24)	0.84 (0.49, 1.44)	0.291
>7.7	22.0 (33.6)	117 (48.0)	135 (33.1)	2.29 (1.53, 3.46)	1.32 (0.79, 2.24)	
Watermelon (g/day)						
<4.5	0.7 (0.8)	46 (18.9)	136 (33.3)	1.00 reference	1.00 reference	
4.5–17.6	10.7 (5.9)	132 (54.1)	137 (33.6)	2.85 (1.90, 4.33)	2.12 (1.31, 3.45)	0.373
>17.6	59.9 (56.1)	66 (27.0)	135 (33.1)	1.45 (0.93, 2.27)	1.27 (0.76, 2.13)	
Citrus fruits (g/day)						
<1.3	0.2 (0.3)	51 (20.9)	134 (32.8)	1.00 reference	1.00 reference	
1.3–6	2.5 (1.3)	133 (54.5)	138 (33.8)	2.53 (1.70, 3.80)	1.74 (1.06, 2.89)	0.713
>6	21.8 (32.4)	60 (24.6)	136 (33.3)	1.16 (0.74, 1.81)	0.91 (0.53, 1.55)	

^†^ ORs were adjusted for education level (primary, high, and tertiary), marital status (married, never married, or separated), smoking habit (never, former, current), prostate cancer in the first-degree relatives (yes, no), life-long physical activity (never, past active, regular), age (year), body mass index (kg/m^2^), ethanol consumption (g/day), and total energy intake (kcal/day); SD: standard deviation; OR: odds ratio; CI: confidence intervals; ^‡^ based on the adjusted models.

## Appendix A

**Table A1:** Associations of the intake of carotenoids and their food sources with the grade of prostate cancer (high: Gleason score 8–10; low-medium: Gleason score ≤ 7).

Nutrient or Food	Reference Group Controls	Grade ^‡^				*p* for Heterogeneity
		Low-Medium	Adjusted OR ^†^ (95% CI)	High	Adjusted OR ^†^ (95% CI)	
Lycopene (μg/day)						
<648	136	37	1.00 reference	40	1.00 reference	
648–1200	137	52	0.65 (0.35, 1.19)	72	1.03 (0.58, 1.86)	
>1200	135	21	0.36 (0.18, 0.7)	22	0.57 (0.29, 1.12)	
*p for linear trend*			0.003		0.104	0.338
α-carotene (μg/day)						
<743	136	38	1.00 reference	51	1.00 reference	
743–976	136	49	1.19 (0.69, 2.06)	56	1.19 (0.7, 2.02)	
>976	136	23	0.72 (0.38, 1.37)	27	0.81 (0.43, 1.5)	
*p for linear trend*			0.322		0.511	0.808
β-carotene (μg/day)						
<3920	137	37	1.00 reference	37	1.00 reference	
3920–5780	135	48	1 (0.57, 1.76)	72	1.92 (1.11, 3.38)	
>5780	136	25	0.6 (0.31, 1.12)	25	0.81 (0.42, 1.57)	
*p for linear trend*			0.118		0.426	0.511
β-cryptoxanthin (μg/day)						
<539	137	28	1.00 reference	34	1.00 reference	
539–867	135	46	1.44 (0.8, 2.62)	54	1.98 (1.1, 3.63)	
>867	136	36	1.11 (0.6, 2.07)	46	1.53 (0.83, 2.84)	
*p for linear trend*			0.737 reference		0.178	0.468
Lutein + zeaxanthin (μg/day)						
<1670	137	35	1.00 reference	34	1.00 reference	
1670–2580	135	49	1.15 (0.66, 2)	73	2.2 (1.26, 3.91)	
>2580	136	26	0.59 (0.31, 1.1)	27	0.88 (0.46, 1.69)	
*p for linear trend*			0.099		0.684	0.388
Tomato (g/day)						
<7.1	137	40	1.00 reference	47	1.00 reference	
7.1–16.5	135	48	0.64 (0.36, 1.14)	66	0.73 (0.42, 1.26)	
>16.5	136	22	0.41 (0.21, 0.77)	21	0.38 (0.19, 0.72)	
*p for linear trend*			0.006		0.003	0.861
Carrot (g/day)						
<1	137	44	1.00 reference	67	1.00 reference	
1–3.2	135	48	0.76 (0.43, 1.32)	46	0.5 (0.29, 0.85)	
>3.2	136	18	0.36 (0.18, 0.69)	21	0.37 (0.2, 0.68)	
*p for linear trend*			0.003		0.002	0.935
Pumpkin (g/day)						
<6.4	137	33	1.00 reference	36	1.00 reference	
6.4–20.5	135	45	0.92 (0.52, 1.63)	56	1.19 (0.67, 2.11)	
>20.5	136	32	0.72 (0.39, 1.32)	42	0.94 (0.52, 1.7)	
*p for linear trend*			0.296		0.834	0.543
Sweet potato (g/day)						
<2.4	137	24	1.00 reference	27	1.00 reference	
2.4–7.7	135	31	0.71 (0.35, 1.43)	45	1.02 (0.52, 2.04)	
>7.7	136	55	1.25 (0.64, 2.43)	62	1.43 (0.73, 2.84)	
*p for linear trend*			0.506		0.304	0.774
Watermelon (g/day)						
<4.5	137	22	1.00 reference	24	1.00 reference	
4.5–17.6	135	56	1.87 (1.01, 3.52)	76	2.43 (1.32, 4.57)	
>17.6	136	32	1.19 (0.62, 2.33)	34	1.33 (0.68, 2.63)	
*p for linear trend*			0.603		0.415	0.819
Citrus fruit (g/day)						
<1.3	137	23	1.00 reference	28	1.00 reference	
1.3–6	135	59	1.6 (0.83, 3.13)	74	1.98 (1.06, 3.76)	
>6	136	28	0.83 (0.42, 1.67)	32	0.93 (0.47, 1.83)	
*p for linear trend*			0.601		0.809	0.654

^‡^ Low-medium grade: Gleason score ≤ 7 and high grade: Gleason score 8–10); ^†^ Adjusted for education level (primary, high, and tertiary), marital status (married, never married, or separated), smoking habit (never, former, current), prostate cancer in the first-degree relatives (yes, no), life-long physical activity (never, past active, regular), age (year), body mass index (kg/m^2^), ethanol consumption (g/day), and total energy intake (kcal/day). CI, confidence interval; OR, odds ratio.
